# S100 proteins in cardiovascular diseases

**DOI:** 10.1186/s10020-023-00662-1

**Published:** 2023-05-22

**Authors:** Yue Zhou, Yiwen Zha, Yongqi Yang, Tan Ma, Hongliang Li, Jingyan Liang

**Affiliations:** 1 Medical College, Yangzhou University, Yangzhou, China; 2 Institute of Translational Medicine, Medical College, Yangzhou University, Yangzhou, 225001 Jiangsu China; 3 Jiangsu Key Laboratory of Integrated Traditional Chinese and Western Medicine for Prevention and Treatment of Senile Diseases, Yangzhou University, Yangzhou, China

**Keywords:** S100 proteins, Cardiovascular diseases, RAGE, TLR-4

## Abstract

Cardiovascular diseases have become a serious threat to human health and life worldwide and have the highest fatality rate. Therefore, the prevention and treatment of cardiovascular diseases have become a focus for public health experts. The expression of S100 proteins is cell- and tissue-specific; they are implicated in cardiovascular, neurodegenerative, and inflammatory diseases and cancer. This review article discusses the progress in the research on the role of S100 protein family members in cardiovascular diseases. Understanding the mechanisms by which these proteins exert their biological function may provide novel concepts for preventing, treating, and predicting cardiovascular diseases.

## Introduction

With the progress in cardiovascular research and the improvement of clinical treatment, human life expectancy has increased (Dwyer-Lindgren et al. 2022). However, cardiovascular diseases continue to be associated with high morbidity and mortality and are one of the major diseases threatening human health and negatively impacting healthcare systems (Tsao et al. 2023). As a result, a large number of basic, translational, and clinical studies in the field of cardiovascular medicine are being undertaken.

S100 proteins have at least 25 identified members in humans, comprising the single largest subcategory of the EF-hand calcium binding superfamily (Marenholz et al. 2004). It is well-documented that many members of the S100 protein family are involved in the onset and progression of cardiovascular diseases (Table 1). Therefore, S100 proteins have great value and potential in the diagnosis, differential diagnosis, treatment, and prognosis of cardiovascular diseases.

**Table 1:** S100 proteins in cardiovascular diseases

S100 proteins	Related cardiovascular diseases
S100A1	Acute myocardial ischemia (Kiewitz et al. 2000; Fan et al. 2019)
	Heart failure (Pleger et al. 2007, 2008, 2011; Desjardins et al. 2009; Belmonte et al. 2011; Soltani et al. 2021; Brinks et al. 2011; Ehlermann et al. 2000)
	Pulmonary hypertension (Teichert-Kuliszewska et al. 2015)
	Myocardial ischemia/reperfusion injury (Jungi et al. 2018)
	Acute coronary syndrome (Fan et al. 2019; Li et al. 2018)
	Acute aortic dissection (Han et al. 2021)
S100A2	Heart failure (Wang et al. 2014b)
S100A4	Acute myocardial infarction (Doroudgar et al. 2016; Gong et al. 2015)
	Unstable angina (Yang et al. 2019)
	Heart failure (Tamaki et al. 2013)
	Myocardial hypertrophy (Sato et al. 2017; Qi et al. 2016; Qian et al. 2018a)
	Viral myocarditis (Wang et al. 2019)
	Atherosclerosis and restenosis of coronary artery lesions (Brisset et al. 2007; Choe et al. 2017; Nagata et al. 2020; Sakic et al. 2022)
S100A5	Atherosclerosis (Awad et al. 2018)
S100A6	Acute myocardial infarction (Cai et al. 2011; Mofid et al. 2017)
	Early adverse left ventricular remodeling after infarction (Tsoporis et al. 2014)
S100A7	Atherosclerosis (Awad et al. 2018)
S100A8/A9	Myocardial infarction (Sreejit et al. 2020; Nagareddy et al. 2020)
	Myocardial ischemia/reperfusion injury (Li et al. 2019)
	Heart failure (Volz et al. 2012; Ma et al. 2012; Wei et al. 2015)
	Autoimmune myocarditis (Otsuka et al. 2009)
	Inflammatory cardiomyopathy (Müller et al. 2017)
	Takayasu arteritis (Goel et al. 2018)
	Acute coronary syndrome (Sakuma et al. 2017; Altwegg et al. 2007; Santilli et al. 2014; Ionita et al. 2010)
	Atherosclerosis (Croce et al. 2009; Eue et al. 2000; Kraakman et al. 2017; Miyamoto et al. 2008; Farris et al. 2011; Hirata et al. 2012; Ionita et al. 2009; McCormick et al. 2005; Maiseyeu et al. 2012)
	Coronary artery disease (Jonasson et al. 2017; Xia et al. 2016)
	Arterial thrombosis (Chen et al. 2018)
	Very late stent thrombosis (Wang et al. 2020a)
	Intracranial aneurysm (Korte et al. 2019, 2020)
	Abdominal aortic aneurysm (Liu et al. 2019)
	Aneurysmal subarachnoid hemorrhage (Wang et al. 2020b)
	Cardiac sarcoidosis (Terasaki et al. 2007)
S100A11	Infective endocarditis (Snipsøyr et al. 2016)
S100A12	Atherosclerosis (Goyette et al. 2009; Scicali et al. 2019; Farokhzadian et al. 2019)
	Familial hypercholesterolemia (Scicali et al. 2019)
	Left ventricular hypertrophy (Yan et al. 2014)
	Aortic valve calcification (Yan et al. 2014)
S100B	Ventricular remodeling after myocardial infarction (Tsoporis et al. 2005b; Mohammadzadeh et al. 2013)
	Cerebral infarction (Garzelli et al. 2020)
	Atrial fibrillation (Zhang et al. 2013; Scherschel et al. 2019)
	Acute coronary syndrome (Cai et al. 2011)
	Dilated cardiomyopathy (Mazzini et al. 2007)
	Heart failure (Li et al. 2011; Chen et al. 2020)
	Myocardial infarction (Tsoporis et al. 2010)
	Atherosclerosis (Wu et al. 2019)
	Cerebral ischemia/reperfusion injury (Yuan et al. 2019)
	Aortic aneurysm (Roberts et al. 2021)
	Cardiac arrest (Deye et al. 2020)

## Structure and functions of S100 proteins

**Fig. 1:**
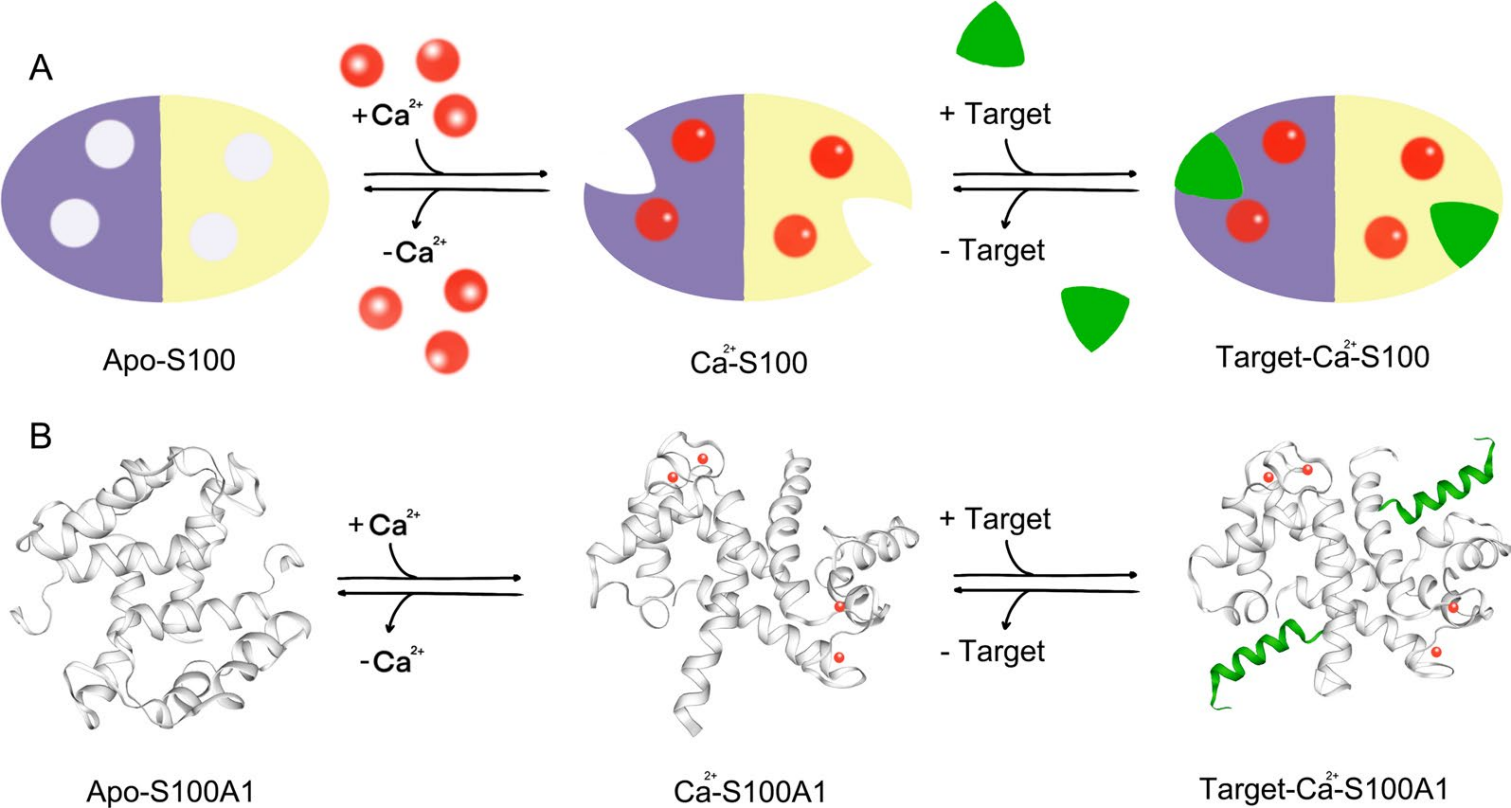
The general structure of S100 proteins. **A** Apo-S100 protein shown with a purple subunit and a yellow subunit. S100 proteins are regulated by Ca^2+^ binding (red circles), which allows them to function as Ca^2+^ sensors that can convert changes in intracellular Ca^2+^ levels into a cellular response. Ca^2+^ binding induces a conformational rearrangement that exposes a hydrophobic cleft, which is necessary for the S100 protein to bind its cellular targets (green) and elicit a physiological response. **B** The mode of S100A1 action demonstrates the calcium-dependent conformational change that is helpful for S100A1 to interact with its target molecules

S100 proteins are small acidic proteins with a molecular weight of 10–12 kDa. In general, the core structural characteristics of S100 proteins are highly conserved, although some sequence variations are present among different members of the family (Martinis et al. 2020). S100 proteins are highly specific and regulate the biological activities of various cells, including cell proliferation, growth, and differentiation, cell cycle regulation, energy metabolism, enzyme activation, transcription, and secretion (Marenholz et al. 2004). All members of the S100 family have two distinct EF-hand conserved functional domains (Fig. 1). The hydrophobic zone on both sides and the hinge zone in the middle form four α-helices, generating a helix-ring-helix ligand structure (Gifford et al. 2007). The carboxy-terminal binding site is usually formed by 12 amino acids, and the amino-terminal binding site is usually composed of 14 amino acids; together, they form a ring structure (Yammani 2012). The carboxy terminal has a high affinity for Ca^2+^. After binding Ca^2+^, the configuration of S100 proteins changes, exposing the hydrophobic fissure which contains the binding site of the target protein, exerting their biological effect by interacting with the target protein (Liu et al. 2013). In addition to Ca^2+^, the functions of many S100 proteins can be regulated by binding zinc or copper ions (Weisz and Uversky 2020).

Most members of the S100 protein family exist as homologous dimers, while a few form heterologous dimers or oligomers, suggesting a diversity of their functions (Wu et al. 2022). S100 proteins have not only intracellular functions but also performs regulatory activities outside the cell, mainly through autocrine or paracrine activation of cell surface receptors such as the late glycation end-product receptor (RAGE) and Toll-like receptor 4 (TLR-4) (Wu et al. 2021). RAGE and TLR-4 are transmembrane receptor proteins and pattern recognition receptors in natural immunity, which mediate the expression of a variety of inflammatory factors and participate in vascular inflammation (Quan et al. 2022; Bai et al. 2016). RAGE and TLR-4 are the receptors of some S100 proteins (Wang et al. 2018); the extracellular parts of RAGE and TLR-4 act as receptors of some S100 proteins and can activate different intracellular signaling pathways, thus mediating the extracellular activity of S100 proteins. Moreover, some S100 proteins can interact with each other in complementary ways. Due of their intimate connection to many human diseases, S100 proteins are being discussed as diagnostic and prognostic biomarkers in Laboratory Medicine and as potential therapeutic targets in the clinical management of patients (Heizmann 2019).

## S100 proteins in cardiovascular diseases

### S100A1

In humans, S100A1 is expressed at the highest level in cardiomyocytes, particularly in the left ventricle, and in vascular endothelial cells. In contrast, its expression in skeletal muscle, brain, and kidney is relatively low. S100A1 is mainly located in the sarcoplasmic reticulum, mitochondria, and myofibrillary segments of cardiomyocytes and is involved in many mechanisms of cardiovascular diseases that are discussed below.

S100A1 regulates calcium homeostasis in cardiomyocytes. S100A1 interacts with sarcoplasmic reticulum calcium pump (SERCA)/phosphoprotein complex in a Ca^2+^-dependent manner, increasing SERCA2 activity and enhancing sarcoplasmic reticulum Ca^2+^ uptake (Stammers et al. 2015). In addition, S100A1 can reduce Ca^2+^ leakage from the sarcoplasmic reticulum during the diastole and increase Ca^2+^ release from the sarcoplasmic reticulum during the systole by regulating the ryanodine receptor 2 (RyR2) (Kettlewell et al. 2005). Abnormal function of RyR2 leads to the continuous loss of Ca^2+^, affects the formation of intracellular Ca^2+^ concentration gradient, promotes spontaneous Ca^2+^ release, and causes membrane potential oscillation. When the amplitude of the oscillation reaches a certain threshold, electrical activity may be triggered, which may be one of the reasons for arrhythmia (Williams et al. 2011). S100A1 protein may help to prevent arrhythmias caused by abnormal Ca^2+^ concentration and reduce the susceptibility of cardiomyocytes to arrhythmia in heart failure by regulating the function of RyR2.

**Fig. 2:**
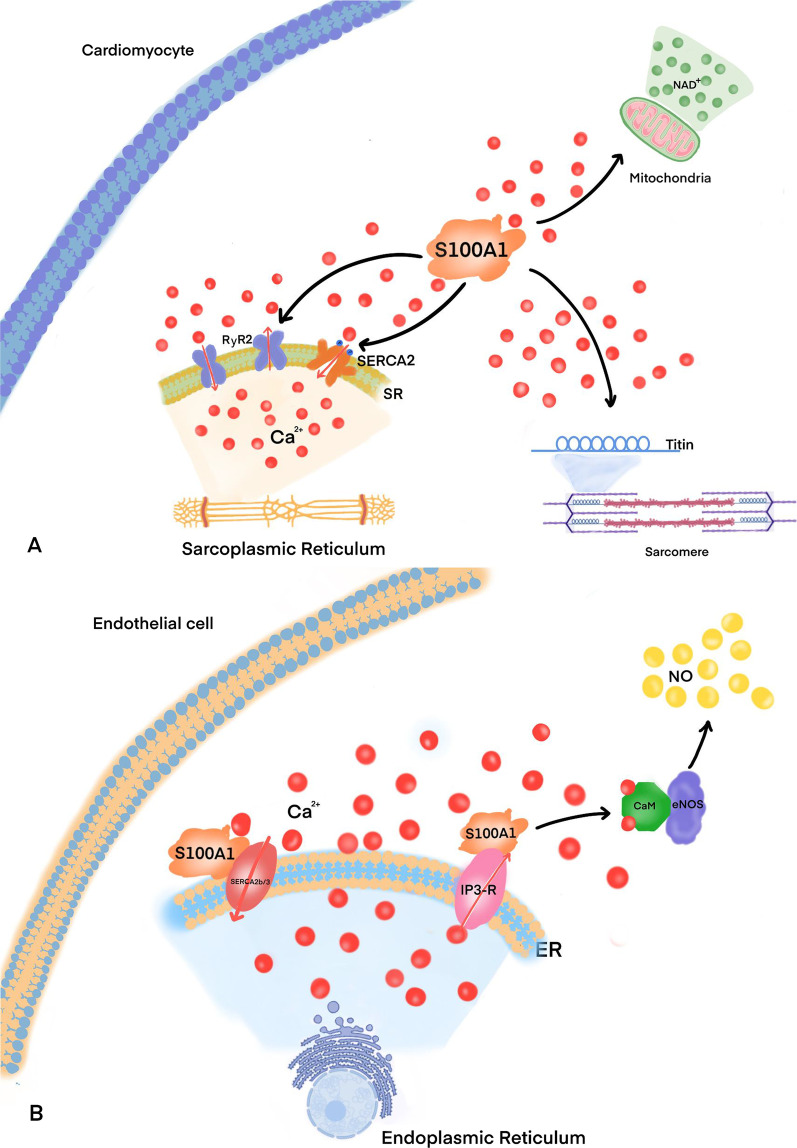
Function of S100A1 in cardiomyocytes and endothelial cells. **A** In cardiomyocytes, S100A1 regulates calcium homeostasis by enhancing SERCA2 activity and controlling the function of RyR2. The regulation of RyR2 by S100A1 reduces Ca^2+^ leakage from the sarcoplasmic reticulum in diastole and increases Ca^2+^ release from the sarcoplasmic reticulum in systole. S100A1 also regulates sarcomere stiffness and Ca^2+^ response. Moreover, S100A1 regulates the mitochondrial function and energy metabolism of cardiomyocytes and inhibits their apoptosis. Finally, S100A1 inhibits ventricular remodeling and prevents arrhythmia. **B** The main role of S100A1 in endothelial cells is the control of Ca^2+^ uptake and release from the endoplasmic reticulum and the regulation of NO balance

In a process mediated by Ca^2+^, myonectin interacts with S100A1 to form “malignant destruction” molecules, which help to prevent myocardial stiffness (Fukushima et al. 2010). Additionally, S100A1 interacts directly with the F1 subunit of mitochondrial ATPase in a Ca^2+^- and pH-dependent manner, regulating mitochondrial function and energy metabolism. S100A1 may increase the activity of citrate dehydrogenase and the production of mitochondrial nicotinamide adenine dinucleotide (NAD^+^) by stimulating the turnover of intracellular Ca^2+^. Moreover, S100A1 may increase ATP synthesis by promoting adenine nucleotide transporter-mediated ATP cytoplasmic translocation. This process permits the adaptation to the increased demand for ATP caused by the increased activity of the sarcoplasmic reticulum and myofilaments (Boerries et al. 2007). S100A1 can also inhibit cardiomyocyte apoptosis (Most et al. 2006) and ventricular remodeling (Fig. 2A) (Pleger et al. 2007; Rohde et al. 2011).

The main role of S100A1 in endothelial cells is the control of Ca^2+^ uptake and release from the endoplasmic reticulum (Most et al. 2013). S100A1 partially co-localizes with SERCA2b and the inositol triphosphate (IP3) receptor (IP3R). IP3 promotes calcium release from the endoplasmic reticulum, while SERCA2b/3 pumps Ca^2+^ back to the endoplasmic reticulum. Calmodulin (CaM) is activated when the cytoplasmic Ca^2+^ concentration is elevated. Activated CaM binds then endothelial NO synthase (eNOS), resulting in the formation of large amounts of NO (Fig. 2B) (Cheriyan et al. 2020). S100A1 knockout results in endothelial dysfunction in vivo (Pleger et al. 2008). Loss of S100A1 leads to pulmonary hypertension associated with endothelial dysfunction and endothelial cell apoptosis (Teichert-Kuliszewska et al. 2015).

Myocardial ischemia is associated with apoptosis and necrosis of cardiomyocytes, and dying cells release S100A1 into the blood (Bertheloot and Latz 2017). Thus, the circulating level of S100A1 can be used as a marker for evaluating acute myocardial ischemia. The concentration of S100A1 in plasma of patients with acute myocardial infarction increased early after infarction peaking at 8 h, and then decreased rapidly with time. No significant change in the serum level of S100A1 was found in angina patients, but some of them overexpressed the MB isoenzyme of creatine kinase (CK-MB) (Kiewitz et al. 2000). Therefore, the serum level of S100A1 has higher specificity as a marker of acute infarction than CK-MB. However, CK-MB has limited cardiac specificity and sensitivity (Bodor 2016). Currently, Cardiac troponin I (cTnI) is considered as the “gold standard” for acute myocardial infarction diagnosis owing to its superior cardiac specificity for cardiac damage, and it shows little or no changes in patients with a skeletal muscle disease or trauma (Danese and Montagnana 2016). Serum cTn concentration increases within 2–4 h after acute myocardial injury, reaches a peak concentration at 10–15 h, and goes back to the baseline level within 5–14 days (Zhang et al. 2022). The sensitivity of circulating S100A1 was superior to that of cTn, because the mean time to elevation was significantly shorter after myocardial reperfusion and S100A1 concentrations decreased faster than cTn and CK-MB (Kiewitz et al. 2000; Völkers et al. 2010; Li et al. 2021). Therefore, the evaluation of S100A1 is beneficial to improve clinical diagnosis and disease progression in patients with acute myocardial infarction. Additionally, elevated serum S100A1 is one of the risk factors for ST-segment elevation, which is associated with cardiac function after myocardial infarction (Fan et al. 2019). S100A1 protein expression is downregulated in heart failure, especially in end-stage heart failure, and is increased in cardiac hypertrophy. In aortic coarctation and myocardial infarction models, S100A1 knockout mice have significantly higher cardiomyocyte apoptosis and more severe ventricular remodeling than wild-type mice. They are also more prone to heart failure and have a lower survival rate (Desjardins et al. 2009).

In heart failure, Ca^2+^ transport in cardiomyocytes is abnormal, with a concentration in systole lower than normal and in diastole higher than normal. These deficiencies indicate that the sarcoplasmic reticulum calcium pump transports Ca^2+^ less efficiently, and its expression and activity are decreased in heart failure cardiomyocytes (Belmonte et al. 2011). Recent studies showed that S100A1 is likely related to the pathogenesis of heart failure (Soltani et al. 2021). S100A1 plays a crucial part in calcium homoeostasis through sarcoplasmic reticulum. Advanced heart failure may change the expression of S100A1 in cardiomyocytes, resulting in lowering S100A1 tissue levels (Imbalzano et al. 2016), and overexpression of S100A1 can effectively improve heart failure (Pleger et al. 2008; Belmonte et al. 2011; Soltani et al. 2021; Brinks et al. 2011; Ehlermann et al. 2000). The gold standard biomarkers for the diagnosis and prognosis of heart failure are B-type natriuretic peptide (BNP) and N-terminal proBNP (NT-proBNP) (Malavolta et al. 2017). Circulating BNP and NT proBNP levels are normally very low, but they are significantly increased in patients with heart failure, while S100A1 expression levels are decreased and positively associated with heart failure severity, progression, and mortality (Ritterhoff and Most 2012). Moreover, adenoviral delivery of S100A1 was effective in the treatment of heart failure in small and large animals and improving the failing function of animal and human cardiomyocytes (Pleger et al. 2007, 2011; Brinks et al. 2011).

Myocardial ischemia–reperfusion injury refers to the pathological process of the aggravation of myocardial damage caused by reperfusion within a certain period of time after partial or complete acute occlusion of coronary artery (Guo et al. 2022). Overexpression of S100A1 has a cardioprotective effect in myocardial ischemia–reperfusion injury and can promote the recovery from global cerebral ischemia–reperfusion injury (Jungi et al. 2018). Brett et al. (2001) studied patients undergoing elective coronary artery bypass graft (CABG) or CABG combined with valve replacement. Confocal laser scanning microscope was used to observe the localization changes of S100A1 in biopsy specimens before and after CPB, and after successful reperfusion 30 min. The results showed that the localization of S100A1 changed significantly at the end of CPB. Reversible repositioning occurred 30 min after successful reperfusion. Confocal microscopy revealed a significant dislocation of S100A1 from cytoplasm to cell surface during myocardial ischemia. This suggests that ischemia-induced release of S100A1 from its dominant position on the contractile fine myofilaments may influence the contraction/relaxation properties of cardiomyocytes during cardiac surgery. By comparing the localization changes of S100A1 subcells before and after CPB, we can predict the occurrence of myocardial reperfusion injury and prepare for the prevention of myocardial ischemia–reperfusion arrhythmia in advance.

More intriguingly, S100A1 exerts opposite regulatory effects in acute coronary syndrome (ACS) (Fan et al. 2019). ACS is a special type of syndrome in coronary heart disease, classified mostly as unstable angina pectoris (UA), acute non-ST segment elevation myocardial infarction (NSTEMI), and acute ST-segment elevation myocardial infarction (STEMI). The first two conditions are collectively called non-ST elevation acute coronary syndrome (NSTE-ACS). Elevated plasma S100A1 concentration in combination with a variety of other biomarkers is an important predictor of STEMI and may reflect cardiac dysfunction after acute coronary ischemia (Fan et al. 2019). S100A1 is also a potential biomarker for predicting the progression of NSTE-ACS, contributing to the diagnosis of early risk stratification and prognosis (Li et al. 2018). Acute aortic dissection is often misdiagnosed as acute myocardial infarction or other cardiovascular disease due to the lack of typical symptoms, leading to delays in treatment. Meanwhile, the diagnosis of acute aortic dissection mainly depends on various imaging technologies, such as magnetic resonance imaging, computed tomography angiography, and ultrasound (Memon et al. 2019). Imaging technologies are relatively time-consuming or unavailable in some hospitals (Carroll et al. 2020; Liu and Huang 2018; Carpenter et al. 2014). Therefore, for the early diagnosis of acute aortic dissection in the clinic. Some markers with high sensitivity and specificity are needed. The plasma concentration of S100A1 in patients with acute aortic dissection is significantly increased, pointing to potential clinical value in diagnosing acute aortic dissection (Han et al. 2021). However, S100A1 has been shown to be an early diagnostic marker for acute myocardial infarction (Kiewitz et al. 2000; Völkers et al. 2010; Li et al. 2021). More further studies are needed to differentiate acute aortic dissection from acute myocardial infarction.

### S100A4

S100A4 is expressed in fibroblasts, keratinocytes, smooth muscle cells (SMCs), cardiomyocytes (Stary et al. 2006), tumor cells, and several other cell types. Inside these cells, the S100A4 expression is strongly related to cell migration and apoptosis (Chow et al. 2017). Besides exerting intracellular function, S100A4 can be secreted as a covalently bound dimer, exerting extracellular functions such as stimulating cell motility, invasion, cytokine production, and promoting inflammation. The well-known function of S100A4 is the induction and promotion of tumor metastasis (Bresnick et al. 2015). S100A4 plays an important role in the body’s inflammatory response, so it can be classified as a damage-associated molecular pattern (DAMP) or an alarmin (O’Dwyer et al. 2016). S100A4 is typically released to the extracellular space from cells in response to stress. It binds to specific immune cell receptors to activate innate immunity and secrete inflammatory mediators, thus promoting the inflammatory response (Barron et al. 2016).

During embryogenesis, S100A4 is secreted by the endoderm wall, promoting the differentiation and proliferation of cardiomyocytes in the early developmental stage and providing an indispensable supporting function in the development of cardiomyocytes (Stary et al. 2006). S100A4 expression protects against myocardial ischemia, supporting cardiac function after myocardial infarction (Doroudgar et al. 2016). In the model of myocardial hypertrophy induced by aortic coarctation or myocardial infarction, S100A4 expression is upregulated, activating extracellular signal-regulated kinase1/2 (ERK1/2). Activated ERK1/2 is involved in the regulation of meiosis, mitosis, cell growth, proliferation, differentiation, migration, and survival (Mutlak and Kehat 2015). Therefore, S100A4 may have a regenerative effect on the injured myocardium (Schneider et al. 2007). Serum S100A4 level was proposed as a novel biomarker for the detection of acute myocardial infarction (Gong et al. 2015). Elevated circulating levels of S100A4 may also be involved in the pathogenesis of unstable angina and may be a predictor of cardiovascular events after the treatment of this condition (Yang et al. 2019).

Tamaki et al. (2013) demonstrated that S100A4 expression was upregulated in a rat model of hypertensive heart disease and, more importantly, S100A4 expression was further increased during the progression to heart failure. However, studies on the role of S100A4 and p53 in myocardial fibrosis revealed that the expression of S100A4 and p21, a downstream gene of p53, in myocardial stromal fibroblasts is regulated by Smad3 and Bmal1 through tumor necrosis factor-α (TNF-α) (Sato et al. 2017). Increased collagen deposition and upregulation of S100A4 and p53 may affect myocardial fibrosis in hypertrophic cardiomyopathy (Qi et al. 2016). Therefore, attenuating myocardial fibrosis may provide therapeutic effects in heart failure and hypertrophy by blocking S100A4. This notion is supported by the documentation that downregulation of S100A4 through the Wnt/β-catenin pathway can alleviate myocardial fibrosis in mice (Qian et al. 2018a). Inhibition of S100A4 and MMP-3 by Ginkgo biloba extract may alleviate viral myocarditis (Wang et al. 2019).

In humans, S100A4 is essentially absent in normal coronary arteries but is expressed in SMCs residing in pulmonary hypertension, atherosclerotic and restenosis coronary artery lesions, and S100A4 is considered a marker of these diseases (Brisset et al. 2007). S100A4 can be synthesized and released from human pulmonary artery smooth muscle cells (hPASMCs) in response to 5-hydroxytryptamine (5-HT). It can then act in an autocrine manner to regulate the proliferation and migration of hPASMCs via activation of RAGE and increase the risk of developing pulmonary arterial hypertension (Lawrie et al. 2005; Dempsie et al. 2011). Vascular smooth muscle cell (VSMC) proliferation can be inhibited by miR-124, which regulates S100A4 (Choe et al. 2017). Interestingly, extracellular S100A4 has a chemotactic effect on SMCs. S100A4 expression is increased in human carotid plaque and positively correlates with the degree of arterial dilation and remodeling (Nagata et al. 2020). Recent studies have shown that the neutralization of extracellular S100A4 reduces the area and necrotic core of atherosclerotic lesions and improves plaque stability (Sakic et al. 2022). The multiple roles of S100A4 in cardiovascular diseases warrant further investigation.

### S100A6

S100A6, also known as calcyclin, regulates the cell cycle, proliferation, and the initiation of apoptosis. The expression of S100A6 in vivo is cell- and tissue-specific. Under baseline physiologic conditions, the expression levels of S100A6 protein differ among cardiomyocytes, endothelial cells, fibroblasts, and main artery SMCs, with fibroblasts having the highest expression and cardiomyocytes the lowest (Tsoporis et al. 2008). However, after the induction of myocardial infarction in rats, the expression of s100A6 was increased due to cardiomyocyte damage (Tsoporis et al. 2005a), and serum level of S100A6 protein was correlated with infarct size (Cai et al. 2011). Since cardiomyocytes are differentiated cells with limited proliferative potential, and apoptosis is an important feature of ischemic myocardial injury in humans (Wei et al. 2020), it is essential to prevent apoptosis to maintain this cell population. TNF-α induces cardiomyocyte apoptosis by the nuclear transcription factor kappa B (NF-қB) pathway, and the overexpression of S100A6 phosphorylates p53 to inhibit cardiomyocyte apoptosis induced by TNF-α (Tsoporis et al. 2008). After ischemia–reperfusion, overexpression of S100A6 attenuates myocardial injury, reduces cell loss, and improves left ventricular systolic function by attenuating apoptosis, myocardial hypertrophy (Tsoporis et al. 2014), and infarct size (Mofid et al. 2017). Thus S100A6 is a potential therapeutic target for the prevention of adverse left ventricular remodeling after infarction.

S100A6 is thought to be involved in cell cycle entry and progression of endothelial cells, and its expression is enhanced in proliferating endothelial cells in reconstructed coronary and carotid arteries (Lerchenmüller et al. 2016). However, no studies on the relationship between S100A6 and VSMCs have been published. The changes in VSMC structure and function are closely related to the occurrence of cardiovascular diseases (Grootaert and Bennett 2021; Xia et al. 2023). During the development of atherosclerosis, abnormal proliferation and migration of VSMCs and the synthesis of the extracellular matrix by these cells are important contributors to early vascular injury (Vacante et al. 2021). Whether S100A6 is involved in the abnormal proliferation and migration of atherosclerotic VSMCs and how this involvement is regulated remains unclear and warrants further research.

### S100A8/A9

S100A8 is usually covalently bound to S100A9, forming an S100A8/A9 heterodimer. Because the complex has a certain antibacterial effect, it is also called calprotectin. S100A8/A9 was first purified from myeloid cells and exhibited inhibition of casein kinase; therefore, it is also known as bone marrow-related protein mainly expressed in the cytoplasm of neutrophils and monocytes. Besides neutrophils and monocytes, the S100A8/A9 complex is also expressed in highly purified B cell populations (Husson et al. 2002), platelets (Healy et al. 2006), cardiomyocytes (Boyd et al. 2008), and SMCs (Inaba et al. 2009). With increasing intracellular Ca^2+^ concentration, S100A8/A9 translocates from the cytoplasm to the cytoskeleton and plasma membrane.

S100A8/A9 plays a primary role in triggering inflammatory response after myocardial infarction since this complex is highly expressed in angiotensin II-stimulated neutrophils, where it accounts for 45% of cytoplasmic protein (Sreejit et al. 2020). Moreover, neutrophils activated by S100A8/A9 after myocardial infarction are implicated in NETosis (Nagareddy et al. 2020), the inflammatory cell death mode of neutrophils. With the death of neutrophils, the intracellular S100A8/9 is released to the extracellular space. The S100A8/A9 heterodimer can induce endothelial barrier function and increase endothelial cell permeability (Wang et al. 2014a), thereby releasing a large number of inflammatory factors.

**Fig. 3:**
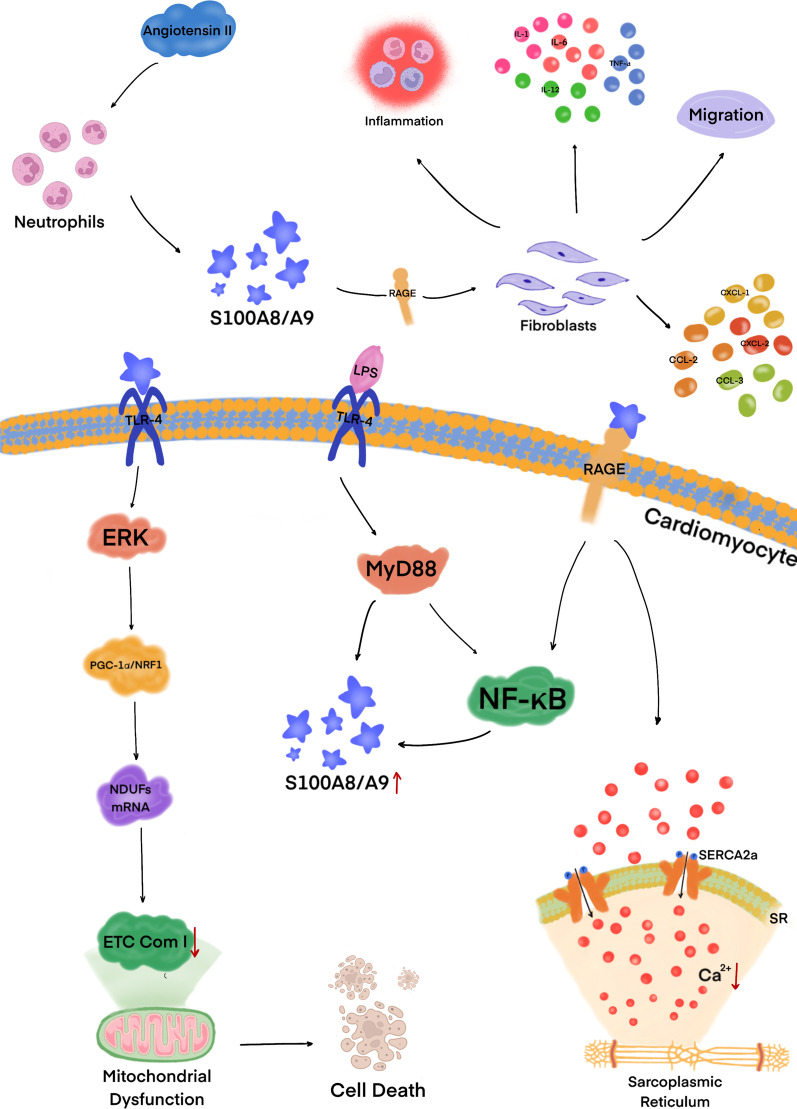
Angiotensin II stimulates neutrophils to produce large amounts of S100A8 and S100A9. The S100A8/A9 complex activates transmembrane RAGE receptors on the surface of cardiac fibroblasts, stimulates cell migration, and activates the inflammatory response by generating significant amounts of cytokines, such as IL-1, IL-6, IL-12, and TNF-α, and chemokines, such as CXCL-1, CXCL-2, CCL-2, and CCL-3. S100A8/A9 activates TLR-4 receptors in cardiomyocytes and inhibits NDUFs genes that promote the formation of ETC complex I by suppressing PGC-1/NRF1. The resulting inhibition of ETC complex I activity causes mitochondrial dysfunction and eventually cell death. In addition, S100A8 and S100A9 reduce Ca^2+^ flux in cardiomyocytes and induce NF-κB expression by inhibiting SERCA2a activity via transmembrane RAGE receptors. Also, lipopolysaccharide activates TLR-4 receptors in cardiomyocytes, resulting in upregulation of S100A8 and S100A9 expression mediated by MyD88 and NF-κB

S100A8/A9 is an autocrine participant in the leukocyte adhesion cascade. S100A8/A9 and TLR-4 are important regulators of the leukocyte recruitment cascade during inflammation in vivo. Lipopolysaccharide activates TLR-4 receptors in cardiomyocytes, resulting in the upregulation of S100A8 and S100A9, which is mediated by myeloid differentiation factor-88 (MyD88) and NF-κB (Pruenster et al. 2015). Takano et al. (2017) established that S100A8 is critical for the activation of NF-қB in T3-induced cardiomyocyte hypertrophy both in vivo and in vitro. In addition, following myocardial infarction, S100A8/A9 downregulates the expression of electron transport chain complex I genes (NDUFs) through the TLR-4/Erk-mediated Pparg coactivator 1 alpha/nuclear respiratory factor 1 signaling pathway. Downregulation of NDUFs inhibits mitochondrial complex I, resulting in mitochondrial respiratory dysfunction of cardiomyocytes and direct myocardial injury (Li et al. 2019). S100A8 and S100A9 also mediate endotoxin-induced cardiomyocyte dysfunction through the RAGE receptor (Boyd et al. 2008). S100A8/A9 exacerbates ischemic heart failure by activating the RAGE-dependent NF-қB signaling pathway (Fig. 3) (Volz et al. 2012). Cardiac overexpression of S100A8/A9 complex may be a self-protective mechanism of inflammation (Ikemoto et al. 2007; Otsuka et al. 2009). It is likely that serum levels of S100A8/A9 complex reflect the severity of heart failure associated with inflammation (Ma et al. 2012). Consistent with these findings, knockout of S100A9 significantly reduces cardiomyocyte death and improves cardiac function, while overexpression of S100A9 in hematopoietic cells aggravates the myocardial ischemia–reperfusion injury (Li et al. 2019). Therefore, S100A8/A9 appears of great significance for the treatment of myocardial infarction and early myocardial ischemia–reperfusion.

The regulation of S100A8/A9 in inflammatory cardiomyopathy became a matter of controversy during the past decade. On the one hand, S100A8/A9 inhibits the inflammatory response in rat autoimmune myocarditis by regulating the pro-inflammatory cytokine network (Otsuka et al. 2009). On the other hand, S100A8 and S100A9 aggravate Coxsackie B3 virus-induced myocarditis (Müller et al. 2017). Thus, more research is needed to confirm these findings and explore the specific mechanisms. S100A8/A9 may become a potential therapeutic target for inflammatory cardiomyopathy.

The S100A8/A9 complex extensively regulates vascular inflammation and participates in response to vascular injury by promoting leukocyte recruitment (Croce et al. 2009). Takayasu arteritis is a chronic non-specific inflammatory vasculitis of unknown pathogeny, involving mainly the aorta and its main branches and the pulmonary arteries, with characteristic of stenotic and occasionally dilated lesions (Wen et al. 2020). Compared with the healthy control group, the expression of TLR4 in Takayasu arteritis patients has an upward trend. The expression of S100A8 and S100A9 as TLR4 ligands is also increased (Kabeerdoss et al. 2019). The activation of TLR-4 may play an important role in the pathogenesis of Takayasu arteritis. S100A8/9 may be an endogenous driver of TLR-4 activation. In Takayasu arteritis, S100A8/9 may have prognostic value as a biomarker of severity and progression of the disease (Goel et al. 2018). But further experiments are needed to confirm this hypothesis.

Elevated concentration of S100A8/9 in coronary artery blood is associated with thrombosis in acute coronary syndrome (ACS) patients and leukocyte activation. S100A8/9 can be used as a unique biomarker of ACS inflammation and thrombosis (Sakuma et al. 2017). Moreover, the concentration of S100A8/A9 in systemic circulation increases before that of markers of myocardial necrosis, making it a prime candidate for detecting unstable plaques and managing ACS (Altwegg et al. 2007). Circulating S100A8/A9 complex in patients with ACS is related to thromboxane-dependent platelet activation, even during low-dose aspirin therapy, suggesting that residual thromboxane contributes to the shedding of S100A8/A9, which may further amplify platelet activation (Santilli et al. 2014). The S100A8/9 complex is emerging as a novel biomarker to distinguish patients with acute coronary syndrome from those with stable coronary heart disease (Ionita et al. 2010).

A cohort study suggested that the dysregulation of cortisol secretion in CAD patients may be associated with an exaggerated pro-inflammatory S100A8/A9 response. Stress-induced S100A8/A9 in CAD patients with impaired cortisol response can enhance the release of this steroid (Jonasson et al. 2017). Arterial thrombosis is associated with an increase in mitogen-activated protein kinase (MAPK) and cyclooxygenase-2 (COX-2) expression via S100A8/A9-activated TLR-4 (Chen et al. 2018). Very late stent thrombosis (VLST) is a rare but serious complication of percutaneous coronary intervention (PCI). The level of 100A8/A9 during VLST is significantly higher than after PCI alone (Wang et al. 2020a). S100A8/A9 is present in the wall and thrombus lumen of enlarged intracranial aneurysms. Given its critical role in vascular inflammation, S100A8/A9 may be used as a biomarker and potential therapeutic target for intracranial aneurysms (Korte et al. 2019). Venous S100A8/A9 level is elevated in both ruptured intracranial aneurysms (rIAs) and unruptured intracranial aneurysms (uIAs), and may be a sign of aneurysm wall inflammation. S100A8/A9 causes macrophage-induced inflammation and vascular wall degeneration, which may explain its higher level in rIAs than in uIAs (Korte et al. 2020). Additionally, the S100A8/A9 complex also contributes to the formation and progression of abdominal aortic aneurysm (Liu et al. 2019). S100A8/A9 may help predict early prognosis and complications of aneurysmal subarachnoid hemorrhage (Wang et al. 2020b).

The S100A8/A9 complex is highly expressed in both human and mouse atherosclerotic lesions (Croce et al. 2009; Eue et al. 2000), and promotes atherosclerosis in diabetic patients (Kraakman et al. 2017). Croce et al. (2009) demonstrated that S100A8/A9 stimulates VSMC proliferation and migration by promoting leukocyte recruitment. In comparison with S100A8/A9-negative monocytes, cells expressing S100A8/A9 preferentially infiltrate atherosclerotic lesions (Eue et al. 2000). The expression of S100A8/A9 complex in neutrophils infiltrating atherosclerotic plaques of unstable angina pectoris is elevated, suggesting its involvement in the inflammation of atherosclerotic plaques in patients with unstable angina pectoris (Miyamoto et al. 2008). Additionally, the urokinase plasminogen activator (uPA)/uPA receptor (uPAR)/plasminogen system and the S100A8/A9 complex interact to accelerate the progression of atherosclerosis (Farris et al. 2011). Moreover, ultrasound showed that type 2 diabetes patients with low carotid plaque density had higher serum S100A8/A9 concentration and increased incidence of cardiovascular complications (Hirata et al. 2012). The expression of S100A9 in human atherosclerotic plaques is consistent with morphological characteristics of increased inflammation and plaque rupture (Ionita et al. 2009). S100A8 and S100A9 in atherosclerotic plaques and calcified stromal vesicles may significantly influence redox- and Ca^2+^-dependent processes in atherosclerosis and its chronic complications, particularly malnutrition calcification (McCormick et al. 2005). Targeting the inflammation-associated S100A8/A9 complex in vivo with antibody-conjugated gadolinium nanoprobes showed plaque enhancement, binding to inflammatory cells, and reduced inflammation. This strategy holds promise as a treatment for atherosclerosis (Maiseyeu et al. 2012). The elevated level of S100A8/9 complex indicates the instability of the atherosclerotic plaque, while the eosinophil cationic protein (ECP) reflects the severity of coronary artery stenosis and predicts the burden of atherosclerosis. These two parameters may become new biomarkers for coronary artery disease (CAD) (Xia et al. 2016). While S100A1 expression levels are particularly high in cardiomyocytes, populations of cardiomyocytes (Boyd et al. 2008), platelets (Healy et al. 2006), and B cells (Husson et al. 2002) that are highly purified exhibit S100A8/A9 expression (Inaba et al. 2009). While both S100A1 and S100A8/A9 play key roles during different phases of CAD, the mechanistic roles that they play are distinct owing to their cell type-specific expression profiles. Both of these proteins are upregulated in cases of acute CAD, but no prior studies have explored potential interactions between these proteins in the context of CAD. Both S100A1 and S100A8/A9 can control energy metabolism and the function of mitochondria (Boerries et al. 2007; Li et al. 2019), suggesting that they may be associated with oxidative stress. The S100A8/A9 complex may also be involved in the pathogenesis of myocardial granuloma, and its serum concentration may be used to diagnose cardiac sarcoidosis (Terasaki et al. 2007).

### S100A12

**Fig. 4:**
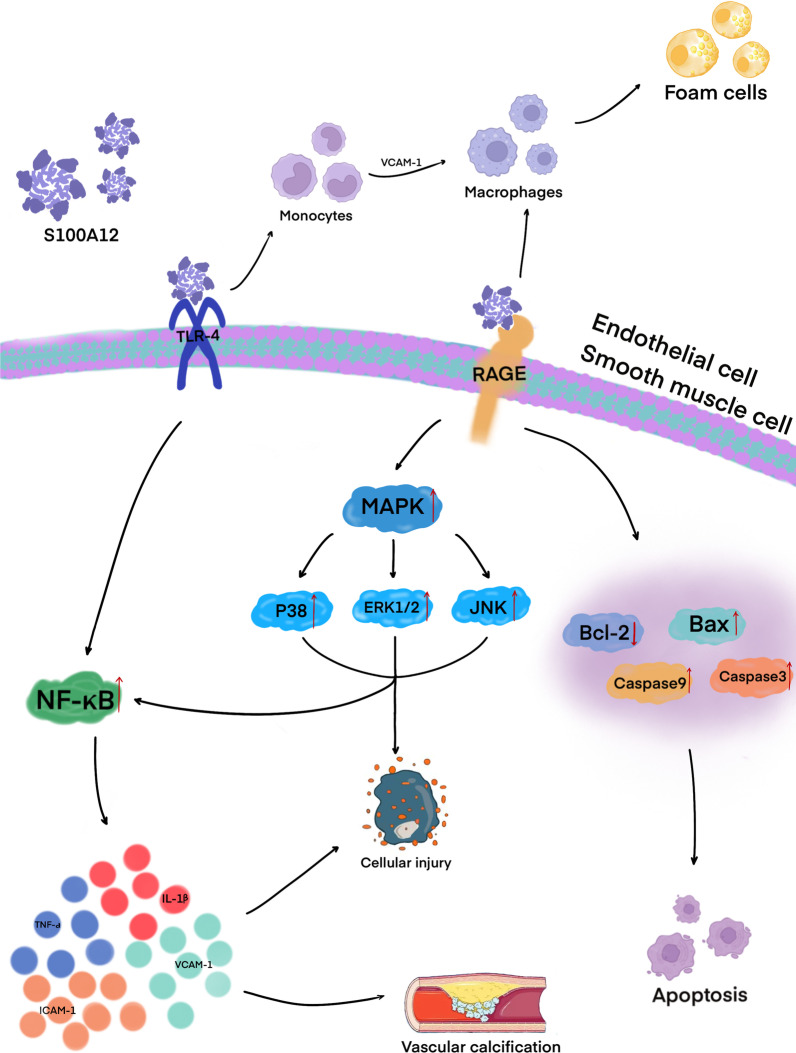
S100A12 is closely related to the onset and development of inflammation. The interaction of S100A12 with RAGE activates inflammatory cells such as macrophages and lymphocytes. When macrophages absorb lipids, they form foam cells that participate in the formation of atherosclerotic lesions. In addition, the interaction of S100A12 with TLR4 activates monocytes and induces an inflammatory response. Activated NF-κB and MAPK pathways produce a large number of pro-inflammatory cytokines (TNF-α, IL-1β) and adhesion molecules (VCAM-1, ICAM-1) and downregulate the expression of the anti-apoptotic protein Bcl-2 and upregulate the expression of pro-apoptotic proteins Bax, caspase-3, and caspase-9, leading to cell apoptosis and vascular calcification

S100A12, like S100A8/A9, is also a bone marrow-related protein. Under physiologic conditions, it is mainly expressed in neutrophils and monocytes/macrophages and can be markedly upregulated in various types of inflammation. S100A12 promotes the onset and development of inflammation by interacting with RAGE and TLR-4. When S100A12 is combined with RAGE, it can activate inflammatory cells such as macrophages and lymphocytes (Xie et al. 2007). In addition, the interaction between human S100A12 and TLR-4 can activate monocytes and inflammatory response (Foell et al. 2013). S100A12 can activate NF-κB and MAPK pathways in VSMCs (Singh et al. 2022), endothelial cells (Zhou et al. 2015) and cardiomyocytes (Xie et al. 2022), which produce a large number of pro-inflammatory cytokines and adhesion molecules, such as TNF-α, interleukin-1β (IL-1β), vascular cell adhesion molecule-1 (VCAM-1), and intercellular adhesion molecule-1 (ICAM-1) (Checconi et al. 2020). S100A12 also can upregulate the expression of vascular endothelial cell adhesion molecules (Das et al. 2012) and activate inflammatory cells and chemotaxis. VCAM-1 induces monocytes to become macrophages and absorb lipids to form foam cells, thus contributing to the formation of atherosclerotic lesions (Fig. 4) (Libby 2006). High levels of S100A12 may alter the cellular microenvironment and promote the formation of atherosclerosis by activating mast cells. However, S100A12 derived from macrophages and foam cells has the opposite effect; it inhibits the rupture of atherosclerotic plaques by suppressing the activity of matrix metalloproteinases (Goyette et al. 2009). S100A12 level is increased in patients with familial hypercholesterolemia and may contribute to a better assessment of cardiovascular risk profile. Since inflammation links hypercholesterolemia to atherosclerosis, S100A12 may help to better define the burden of atherosclerosis in patients with high cholesterol levels (Scicali et al. 2019). In addition, S100A12 binds to CD36 protein with high affinity. CD36 plays an important role in fatty acid absorption and the expression of CD36 is regulated by RAGE and TLR-4. These observations suggest that the S100A12-CD36 axis may play a role in the pathogenesis of atherosclerosis (Farokhzadian et al. 2019).

S100A12 is not expressed in wild mice. Transgenic mice targeted to express human S100A12 in VSMCs develop thoracic aneurysms (Hofmann Bowman et al. 2010). Reduction of S100A12 or its receptor RAGE in SMCs can reduce cell apoptosis and decrease caspase 3 activation (Das et al. 2012). Follow-up researches find that S100A12 is markedly expressed in the tissue and serum of patients with thoracic aortic aneurysms and dissections. Therefore, elevated S100A12 level could play a vital role in predicting preoperative complications in patients with thoracic aortic aneurysms and dissections (Jiang et al. 2014). Vascular calcification is a pathological condition occurring in atherosclerosis. The expression of S100A12 in VSMCs induces calcification conversion of the atherosclerotic plaques in apolipoprotein E deficient mice, which will lead to plaque morphologies considered more vulnerable (Hofmann Bowman et al. 2011). The S100A12-mediated inflammatory response intensifies left ventricular hypertrophy and aortic valve calcification in mice with chronic renal insufficiency in a RAGE-dependent manner (Yan et al. 2014). Moreover, serum S100A12 is upregulated in patients with ACS (Buyukterzi et al. 2017). Hence, S100A12 can be used as a marker of coronary plaque instability. By activating ERK signaling pathway, S100A12 promotes inflammation, oxidative stress, and apoptosis induced by oxygen–glucose deprivation and reperfusion (OGD/R) (Zhang et al. 2020). It decreases the expression of anti-apoptotic protein Bcl-2 and increases the expression of pro-apoptotic proteins Bax, caspase-3, and caspase-9 (Fig. 3). In patients with intracerebral hemorrhage, a higher level of S100A12 is positively correlated with inflammation, bleeding severity, and short-term mortality, suggesting that S100A12 may be a biomarker for predicting poor outcomes of hemorrhagic stroke (Qian et al. 2018b).

S100A12 level is also elevated in pulmonary hypertension patients and is positively associated with mortality. In these patients, S100A12 can be used as a predictor of cardiac output deterioration and mortality (Tzouvelekis et al. 2018). Pulmonary hypertension is a progressive and fatal disease, characterized by dysfunction of pulmonary artery endothelial cells and abnormal proliferation of pulmonary artery SMCs, leading to vascular structure remodeling, progressive occlusion of vascular lumen, increased pulmonary vascular resistance, and eventually leading to right heart failure and death (Liu et al. 2022). Pulmonary hypertension is known as a potential complication of most cardiovascular and respiratory diseases (Garcia-Ribas et al. 2021), and it has been linked to significantly increased mortality rates (Reque et al. 2016). S100A12 level is elevated in pulmonary hypertension patients, so it is positively associated with mortality. In these patients, S100A12 can be used as a predictor of cardiac output deterioration and mortality (Tzouvelekis et al. 2018). Stress activation of MAPK pathway including p38 is related to the pathogenesis of pulmonary hypertension. Elevated levels of phosphorylated p38 have been observed in the pulmonary vascular system of patients with idiopathic pulmonary hypertension (MacNee et al. 2013), resulting in reversal of pulmonary hypertension in two experimental models of pulmonary hypertension by inhibiting the p38 signaling pathway with selective antagonists (Church et al. 2015). As mentioned above, S100A12 with its receptor RAGE induces activation of several adaptor molecules involved in NF-κB and MAPK pathways. These two pathways are closely related to cell survival and proliferation (Secchiero et al. 2003). S100A12 may worsen VSMC proliferation and survival or endothelial dysfunction in pulmonary hypertension via MAPK or NF-κB signaling pathways, and even lead to increased mortality.

Kawasaki disease is an acute febrile idiopathic disease with systemic inflammatory lesions of small and medium arteries as the main pathological manifestation. These lesions can lead to long-term cardiac complications, such as coronary artery dilation or injury and even a concurrent aneurysm. At present, the diagnosis of Kawasaki disease mainly depends on clinical symptoms, while the coronary aneurysm is diagnosed by ultrasound examination. Serum S100A12 is elevated in acute Kawasaki disease. Meijer et al. (2012) established that the increase in circulating S100A12 is not only related to the stage of the disease but also associated with the response to treatment in children with acute Kawasaki disease. In addition, S100A12 may indicate the presence of a coronary artery injury in children with Kawasaki disease. For example, Fu et al. (2010) documented that in Kawasaki disease patients with coronary artery injury, serum S100A12 level is increased acutely but then stabilizes and gradually decreases in the convalescence stage. Therefore, it is believed that the expression of S100A12 may indicate the presence of coronary artery injury in children affected by Kawasaki disease or may even be involved in the mechanism of coronary artery injury. It has recently been confirmed that S100A12, as a highly expressed vector of aseptic inflammation in Kawasaki disease, may activate human coronary artery endothelial cells (hCAECs) in an IL-1 β-dependent manner. These findings data advance our understanding of the role of S100A12 and IL-1β in the mechanism of pathogenesis of Kawasaki disease (Armaroli et al. 2019).

### S100B

S100B is a protein with multiple functions in inflammation and cell damage. It is also an alarm protein that acts as a danger signal. S100B is involved in various diseases, such as neurological disorders, where cellular stress and immune activation play a key role. S100B is mainly expressed in astrocytes, oligodendrocytes, and Schwann cells (Heizmann et al. 2002) and exerts both intracellular and extracellular effects regulating cell proliferation, apoptosis, energy metabolism, and other processes. Under physiological conditions, S100B is not expressed in cardiomyocytes but can be expressed under ischemic hypoxia and in the peri-infarct region after myocardial infarction (Jönsson et al. 2001). Myocardial injury and impairment of cardiac pump function can cause cerebral hypoperfusion, leading to hypoxic brain injury. To exclude the influence of the brain on S100B expression, Mazzini et al. (2005) performed ischemia–reperfusion of the heart ex vivo using the Langendorff technique. Langendorff isolated heart perfusion technology realizes coronary artery retrograde perfusion by inserting a catheter into the heart aorta, which can be used to evaluate the systolic function of the heart and coronary artery characteristics without neurohumoral regulation (Ye and Chen 2015). The level of S100B reached its peak after 20 min of ischemia and rapidly decreased to normal value after reperfusion, suggesting that ischemia and hypoxia are responsible for the induction of S100B expression in cardiomyocytes.

S100B protein has been implicated in many cardiovascular diseases. S100B can negatively regulate myocardial hypertrophy (Parker et al. 1998), promote cell apoptosis, and participate in ventricular remodeling after myocardial infarction (Tsoporis et al. 2005b). In addition, the expression of S100B may regulate cardiac metabolic function in the remodeling of the post-infarcted diabetic heart (Mohammadzadeh et al. 2013). Moreover, secondary infarction caused by the rupture of cerebral arteriovenous malformation can cause secondary elevation of serum S100B protein level (Garzelli et al. 2020).

Analysis of the expression of atrial proteins in patients with chronic atrial fibrillation by proteomics and liquid chromatography showed that the upregulation of S100B expression may be involved in the repair of tissue injury and adaptive remodeling by regulating the synthesis of fibroblast growth factor and platelet-derived growth factor (Zhang et al. 2013). In addition, myofibroblast proliferation is induced by secreted vascular endothelial growth factor (VEGF) by cardiomyocytes in response to S100B via RAGE ligation, which may help promote tissue damage repair and adaptive remodeling in patients with chronic atrial fibrillation (Tsoporis et al. 2012). After catheter ablation of atrial fibrillation, cardiac glial cells release S100B, which acts as a neurotrophic substance (Scherschel et al. 2019). Serum S100B level is also significantly increased in patients with acute coronary syndrome (Cai et al. 2011). In dilated cardiomyopathy, the expression of S100B is significantly correlated with N-terminal B-type natriuretic peptide precursor (NT-proBNP), and the combination of the two can be used to evaluate the systolic function of the heart (Mazzini et al. 2007). The level of S100B in patients with chronic heart failure is significantly increased and significantly correlates with left ventricular ejection fraction, left ventricular end-diastolic volume, and NT-proBNP. At 1-year follow-up, the incidence of major cardiovascular events was higher in heart failure patients with increased S100B levels (Li et al. 2011). These results indicate that S100B is significantly correlated with cardiac function and prognosis of patients with cardiac insufficiency. Additionally, single nucleotide polymorphism (SNP) of S100B rs9722 is associated with the risk of chronic heart failure in the Chinese Han population (Chen et al. 2020).

**Fig. 5:**
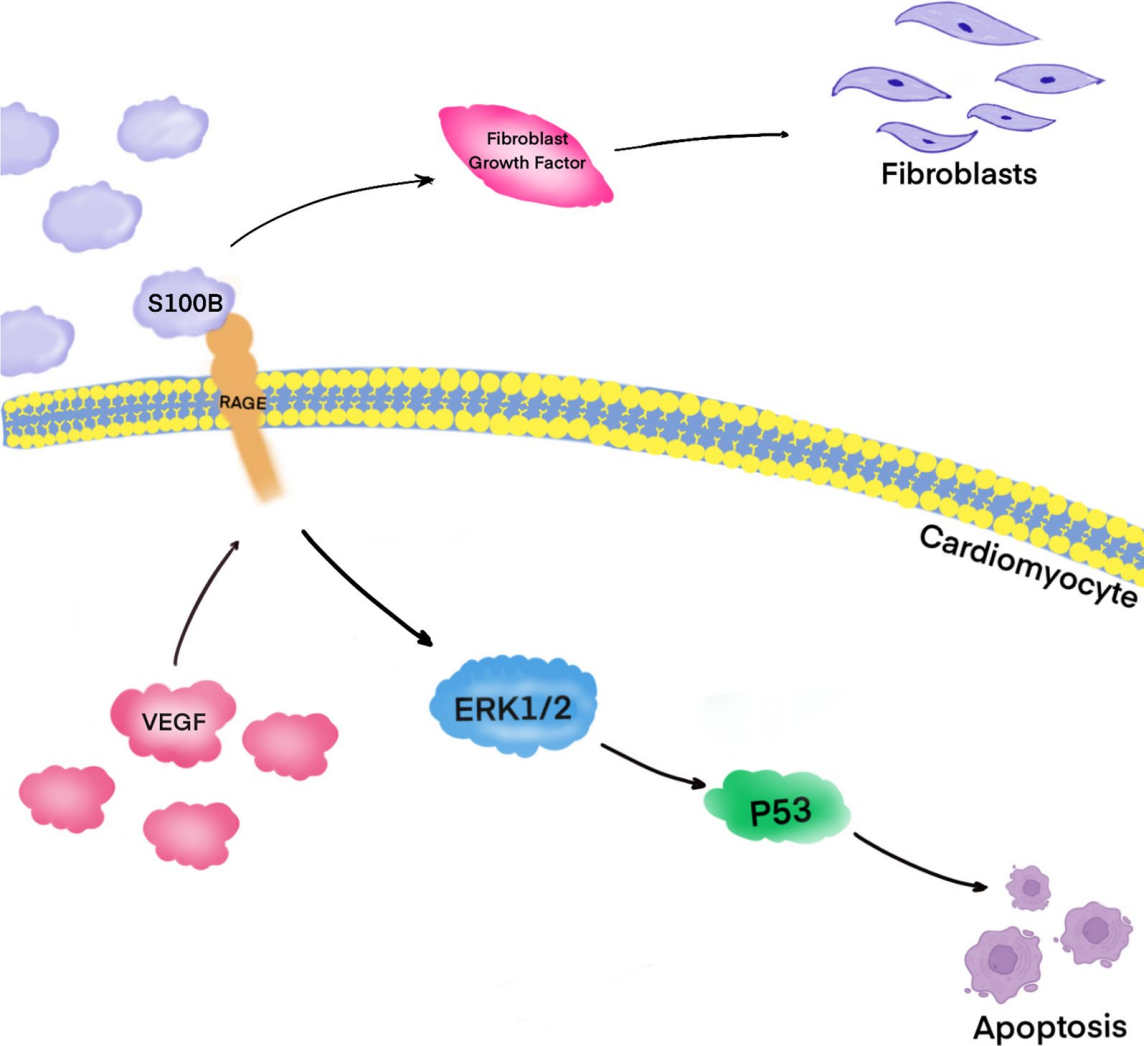
VEGF secreted by cardiomyocytes activates the response of the RAGE receptor to S100B, promotes the release of fibroblast growth factor, and induces the proliferation of cardiac fibroblasts. In addition, the interaction between S100B and RAGE activates p53 and initiates apoptosis through the MEK-ERK1/2 pathway

S100B also has extracellular regulatory activity. In contrast to extracellular S100A1, which inhibits cardiomyocyte apoptosis, S100B is released into plasma after myocardial infarction and regulates apoptosis through extracellular mechanisms. In cultured cardiomyocytes, exogenously administered S100B interacts with RAGE, inducing the release of mitochondrial cytochrome C, and triggering apoptosis through ERK1/2 and p53 signaling pathways (Fig. 5). S100B can also stimulate the secretion of VEGF by cardiomyocytes in a RAGE-dependent manner to induce scar formation in the infarcted area (Tsoporis et al. 2010, 2012). S100B is also involved in atherosclerosis; it triggers the formation of neovascularization in damaged arteries in rats by maintaining a balance between double-positive Sca-1^+^/CXCR4^+^ progenitors and vascular SMCs. This balance is associated with direct activation of RAGE and indirect induction of SDF-1α by PI3K/AKT and NF-κB pathways (Wu et al. 2019). Recently, many studies have shown that S100B can be used as a biomarker to evaluate the improvement of reperfusion after carotid artery stenting (Yuan et al. 2019) and the recovery after intervention in patients that underwent cardiac surgery (Imbalzano et al. 2020), to detect the damage of the blood–brain barrier after thoracic and abdominal aortic aneurysm repair (Roberts et al. 2021), and to predict imminent cardiac arrest (Deye et al. 2020).

### Others

In addition to the S100 proteins reviewed above, some other members of this large family are associated with cardiovascular diseases. For example, Wang et al. (2014b) demonstrated that S100A2 can positively regulate intracellular calcium release and reuptake. It increases cardiac Ca^2+^ transients and contractility and enhances Ca^2+^ release from the sarcoplasmic reticulum via RyR. Thus, the systolic and diastolic functions of cardiomyocytes are augmented. The ectopic expression of S100A2 can effectively improve the defective contractility of failing cardiomyocytes. In contrast, S100A6, despite its high homology with S100A2, does not affect cardiomyocyte Ca^2+^ transients or systolic performance. Awad et al. (2018) found that S100A7 and S100A15 are involved in the upregulation of pro-inflammatory proteins in psoriasis and are associated with intima-media thickness of the left and right common carotid arteries, highlighting the importance of their role in atherosclerosis. Another study found that S100A11, together with other indices, such as circulating cardiac troponin, could be used as a biomarker to diagnose infective endocarditis (Snipsøyr et al. 2016). Therefore, more studies are needed to elucidate the relationship between these members of the S100 family and cardiovascular diseases.

## Conclusion

In summary, S100 proteins are not merely inflammatory markers but also participants in the onset and development of cardiovascular diseases. Understanding their functions is of great value to future studies of cardiovascular diseases. However, although significant changes in their expression take place at the onset of disease, the specific mechanism of action of each member protein is substantially different. In addition, the use of S100 proteins as therapeutic targets for cardiovascular disease presents a particular challenge, as they are associated with many mechanisms of inflammation and cell and tissue injury. To identify specific targets that could be used in prospective clinical trials, the biological characteristics and regulatory mechanism of S100 proteins in cardiovascular diseases need to be further explored.

## Data Availability

Not applicable.
